# Safety Monitoring of an Additional Dose of COVID-19 Vaccine — United States, August 12–September 19, 2021

**DOI:** 10.15585/mmwr.mm7039e4

**Published:** 2021-10-01

**Authors:** Anne M. Hause, James Baggs, Julianne Gee, Paige Marquez, Tanya R. Myers, Tom T. Shimabukuro, David K. Shay

**Affiliations:** 1 CDC COVID-19 Response Team.

On August 12, 2021, the Food and Drug Administration (FDA) amended Emergency Use Authorizations (EUAs) for the Pfizer-BioNTech and Moderna COVID-19 vaccines to authorize administration of an additional dose after completion of a primary vaccination series to eligible persons with moderate to severe immunocompromising conditions (*1*,*2*). On September 22, 2021, FDA authorized an additional dose of Pfizer-BioNTech vaccine ≥6 months after completion of the primary series among persons aged ≥65 years, at high risk for severe COVID-19, or whose occupational or institutional exposure puts them at high risk for COVID-19 (*1*). Results from a phase 3 clinical trial conducted by Pfizer-BioNTech that included 306 persons aged 18–55 years showed that adverse reactions after receipt of a third dose administered 5–8 months after completion of a 2-dose primary mRNA vaccination series were similar to those reported after receipt of dose 2; these adverse reactions included mild to moderate injection site and systemic reactions (*3*). CDC developed v-safe, a voluntary, smartphone-based safety surveillance system, to provide information on adverse reactions after COVID-19 vaccination. Coincident with authorization of an additional dose for persons with immunocompromising conditions, the v-safe platform was updated to allow registrants to enter information about additional doses of COVID-19 vaccine received. During August 12–September 19, 2021, a total of 22,191 v-safe registrants reported receipt of an additional dose of COVID-19 vaccine. Most (97.6%) reported a primary 2-dose mRNA vaccination series followed by a third dose of the same vaccine. Among those who completed a health check-in survey for all 3 doses (12,591; 58.1%), 79.4% and 74.1% reported local or systemic reactions, respectively, after dose 3, compared with 77.6% and 76.5% who reported local or systemic reactions, respectively, after dose 2. These initial findings indicate no unexpected patterns of adverse reactions after an additional dose of COVID-19 vaccine; most of these adverse reactions were mild or moderate. CDC will continue to monitor vaccine safety, including the safety of additional doses of COVID-19 vaccine, and provide data to guide vaccine recommendations and protect public health.

V-safe is a voluntary, smartphone-based U.S. safety surveillance system; vaccinated persons eligible to receive authorized or licensed vaccine product may register in v-safe. The v-safe platform allows existing registrants to report receiving an additional dose of COVID-19 vaccine and new registrants to enter information about all doses of COVID-19 vaccine received. V-safe health surveys are sent during days 0–7 after each dose of vaccine and include questions about local injection site and systemic reactions and health impacts.* Surveys are sent for the most recent dose entered.^†^ Staff members from the Vaccine Adverse Event Reporting System (VAERS) contact registrants who indicate that medical attention was sought after vaccination and encourage or facilitate completion of a VAERS report, if indicated.^§^

Among v-safe registrants who reported receipt of an additional COVID-19 vaccine dose during August 12–September 19, 2021, demographic data, local and systemic reactions, and health impacts reported during days 0–7 were described by pattern of vaccination (i.e., manufacturer of vaccine received for each dose). Persons who reported receiving a primary series from different manufacturers or a manufacturer that was unknown or unavailable in the United States, or 2 doses of vaccine after receipt of a Janssen (Johnson & Johnson) single-dose vaccine (150) were excluded from the analysis of adverse reactions after receipt of the additional dose. Time elapsed from completion of the primary vaccination series to receipt of an additional dose was described by pattern of vaccination. Adverse event profiles after doses 2 and 3 were compared for registrants who received mRNA vaccine from the same manufacturer for all 3 doses.^¶^ SAS software (version 9.4; SAS Institute) was used to conduct all analyses. These surveillance activities were reviewed by CDC and conducted consistent with applicable federal law and CDC policy.**

During August 12–September 19, 2021, a total of 22,191 v-safe registrants reported receipt of an additional dose of COVID-19 vaccine after completing the primary series (Table 1). Among these, 14,048 (63.3%) were female, and approximately 30% each were aged 18–49, 50–64, and 65–74 years. Most registrants (21,662; 97.6%) reported that they received a third dose from the same manufacturer as their primary mRNA vaccine series, including 98.6% of Moderna recipients and 98.2% of Pfizer-BioNTech recipients. Few registrants (341; 1.5%) reported a primary mRNA vaccine series followed by an additional dose of mRNA vaccine from a different manufacturer, a dose of Janssen vaccine after receipt of a primary mRNA vaccination series (10; 0.05%), or an additional dose of COVID-19 vaccine from any manufacturer after Janssen vaccine (178; 0.8%).

**TABLE 1:** Demographic characteristics of persons who received an additional dose of COVID-19 vaccine (N = 22,191)* and completed at least one v-safe health check-in survey on days 0–7 after vaccination, by primary vaccination series and manufacturer of subsequent dose received — United States, August 12–September 19, 2021

Characteristic	Moderna, %^†^ (n = 10,601)			Pfizer-BioNTech, %^†^ (n = 11,412)			Janssen, %^†,§^ (n = 178)			Total (N = 22,191)
	Dose 3 Moderna (n = 10,453; 98.6%)	Dose 3 Pfizer-BioNTech (n = 144; 1.4%)	Dose 3 Janssen (n = 4; 0.04%)	Dose 3 Pfizer-BioNTech (n = 11,209; 98.2%)	Dose 3 Moderna (n = 197; 1.7%)	Dose 3 Janssen (n = 6; 0.1%)	Dose 2 Janssen (n = 48; 27.0%)	Dose 2 Moderna (n = 64; 36.0%)	Dose 2 Pfizer-BioNTech (n = 66; 37.1%)	
**Sex**										
Female	63.8	63.9	50.0	63.0	63.5	33.3	39.6	57.8	59.1	**63.3**
Male	35.1	34.0	50.0	36.1	36.0	66.7	60.4	42.2	40.9	**35.7**
Unknown	1.0	2.1	0	0.9	0.5	0	0	0	0	**1.0**
**Age group, yrs**										
0–17	0.0	0.7	0.0	0.6	0.0	0.0	0.0	0.0	0.0	**0.3**
18–49	25.7	36.1	25.0	31.5	42.6	50.0	54.2	60.9	57.6	**29.1**
50–64	28.4	27.1	50.0	31.1	29.9	0.0	33.3	34.3	30.3	**29.8**
65–74	33.9	27.1	0.0	27.8	21.3	50.0	10.4	4.7	9.1	**30.5**
75–84	10.9	9.0	25.0	8.3	5.6	0.0	2.1	0.0	3.0	**9.5**
≥85	1.1	0.0	0.0	0.7	0.5	0.0	0.0	0.0	0.0	**0.9**
**Ethnicity**										
Hispanic/Latino	8.0	15.3	0	8.2	5.6	0	25.0	6.3	10.6	**8.2**
Non-Hispanic/Latino	87.7	81.9	100	87.6	90.9	100	54.2	89.1	89.4	**87.6**
Unknown	4.3	2.8	0	4.2	3.6	0	20.8	4.7	0	**4.2**
**Race**										
AI/AN	0.5	0.7	0	0.5	0.5	0	2.1	0	0	**0.5**
Asian	4.9	5.6	0	6.1	7.1	0	2.1	14.1	13.6	**5.6**
Black	5.6	3.5	0	6.2	1.5	16.7	6.3	6.3	9.1	**5.9**
NHPI	0.2	0	0	0.3	0.5	0	4.2	0	0	**0.3**
White	82.6	82.6	100	80.4	85.8	66.7	56.3	71.9	69.7	**81.4**
Multiracial	1.9	2.1	0	1.8	1.5	16.7	4.2	4.7	3.0	**1.9**
Other	2.1	4.2	0	2.1	0.5	0	6.3	1.6	3.0	**2.1**
Unknown	2.3	1.4	0	2.5	2.5	0	18.8	1.6	1.5	**2.4**

**Abbreviations:** AI/AN = American Indian/Alaska Native; NHPI = Native Hawaiian or other Pacific Islander.

* Percentage of registrants who completed at least one v-safe health check-in survey on days 0–7 after vaccination.

^†^ Primary vaccination series.

**^§^** Includes persons who received a primary Janssen single-dose and 1 additional dose of vaccine from the listed manufacturers.

Among the 22,191 v-safe registrants, the median interval from completion of the primary COVID-19 vaccination series to receipt of an additional dose was 182 days (interquartile range [IQR] = 160–202 days) (Table 2). Among those who received 2 doses of Janssen vaccine, the median interval between doses was shorter (84 days; IQR = 16–136 days).

**TABLE 2:** Adverse reactions reported by persons who received an additional dose of COVID-19 vaccine (N = 22,191)* and completed at least one v-safe health check-in survey on days 0–7 after vaccination, by primary vaccination series and manufacturer of subsequent dose received — United States, August 12–September 19, 2021

Reaction	Moderna, %^†^ (n = 10,477)			Pfizer-BioNTech, %^†^ (n = 11,284)			Janssen, %^†,§^ (n = 174)			Total (N = 22,191)
	Dose 3 Moderna (n = 10,453; 98.6%)	Dose 3 Pfizer-BioNTech (n = 144; 1.4%)	Dose 3 Janssen (n = 4; 0.04%)	Dose 3 Pfizer-BioNTech (n = 11,209; 98.2%)	Dose 3 Moderna (n = 197; 1.7%)	Dose 3 Janssen (n = 6; 0.1%)	Dose 2 Janssen (n = 48; 27.0%)	Dose 2 Moderna (n = 64; 36.0%)	Dose 2 Pfizer-BioNTech (n = 66; 37.1%)	
**Days since primary series, median (IQR)**	182 (164–198)	183 (161–204)	173 (141–182)	183 (157–209)	186 (161–217)	123 (113–182)	84 (16–136)	156 (140–164)	150 (136–167)	**182 (160–202)**
**Any injection site reaction**	**80.9**	**64.6**	**75.0**	**69.4**	**81.7**	**83.3**	**25.0**	**70.3**	**80.3**	**74.9**
Itching	20.0	11.8	0	8.4	10.2	16.7	10.4	6.3	7.6	**13.9**
Pain	75.9	60.4	75.0	66.6	80.2	83.3	20.8	68.8	74.2	**71.0**
Redness	25.2	8.3	0	9.8	20.8	16.7	6.3	7.8	12.1	**17.1**
Swelling	33.6	17.4	0	16.8	30.5	16.7	6.3	12.5	18.2	**24.8**
**Any systemic reaction**	**75.2**	**59.7**	**50.0**	**65.1**	**76.1**	**100**	**31.3**	**68.8**	**63.6**	**69.9**
Abdominal pain	8.4	3.5	0	6.4	8.1	16.7	4.2	3.1	6.1	**7.3**
Myalgia	49.8	29.2	0	36.3	49.2	50.0	20.8	45.3	33.3	**42.7**
Chills	31.3	8.3	50.0	17.5	33.5	50.0	8.3	23.4	10.6	**24.1**
Diarrhea	9.9	7.6	0	9.0	9.6	16.7	8.3	6.3	9.1	**9.4**
Fatigue	61.8	44.4	0	51.0	60.9	83.3	14.6	48.4	50.0	**56.0**
Fever	36.4	20.1	50.0	22.2	37.1	50.0	6.3	37.5	12.1	**29.0**
Headache	49.0	31.1	0	38.4	49.7	83.3	18.8	35.9	40.9	**43.4**
Joint pain	33.0	18.8	0	23.0	31.0	33.3	16.7	20.3	19.7	**27.7**
Nausea	18.8	10.4	25.0	13.6	21.3	33.3	8.3	9.4	18.2	**16.1**
Rash	2.3	0.7	0	1.9	2.5	0	4.2	1.6	1.5	**2.1**
Vomiting	2.2	2.1	25.0	1.4	2.0	0	2.1	0	0	**1.7**
**Any health impact**	**39.2**	**19.4**	**0**	**25.2**	**39.1**	**33.3**	**16.7**	**28.1**	**24.2**	**31.8**
Unable to perform normal daily activities	35.2	18.1	0	22.1	33.0	33.3	10.4	25.0	15.2	**28.3**
Unable to work or attend school	13.7	4.9	0	9.0	21.3	16.7	10.4	6.3	13.6	**11.3**
Needed medical care	2.1	1.4	0	1.5	3.0	0	6.3	0	0	**1.8**
Telehealth	0.9	0.7	0	0.7	1.0	0	2.1	0	0	**0.8**
Clinic	0.7	0.7	0	0.6	0.5	0	4.2	0	0	**0.6**
Emergency visit	0.2	0	0	0.2	0	0	4.2	0	0	**0.2**
Hospitalization	0.05	0	0	0.1	0	0	0	0	0	**0.1**

**Abbreviation:** IQR = interquartile range.

* Percentage of registrants who completed at least one v-safe health check-in survey on days 0–7 after vaccination.

^†^ Primary vaccination series.

**^§^** Includes persons who received a primary Janssen single-dose and one additional dose of vaccine from the listed manufacturers.

Local (16,615; 74.9%) and systemic (15,503; 69.9%) reactions were frequently reported during the week after an additional dose of COVID-19 vaccine, most commonly on the day after vaccination. Frequently reported reactions were injection site pain (15,761; 71.0%), fatigue (12,429; 56.0%), and headache (9,636; 43.4%).

Among 22,191 additional dose recipients, a total of 7,067 (31.8%) reported health impacts, and approximately 28.3% (6,287) reported they were unable to perform normal daily activities, most commonly on the day after vaccination. Medical care was sought by 401 (1.8%) registrants, and thirteen (0.1%) were hospitalized. Reasons for receiving medical care or hospitalization were not identified in the v-safe survey; however, registrants who indicate that medical attention was sought after vaccination are contacted by VAERS staff and encouraged to complete a VAERS report.

Among 21,658 v-safe registrants who received the same mRNA vaccine for all 3 doses, 12,591 (58.1%) completed at least one health check-in survey on days 0–7 after all 3 doses; 79.4% and 74.1% reported local or systemic reactions, respectively, after dose 3, compared with 77.6% and 76.5% who reported local or systemic reactions, respectively, after dose 2. Among registrants who received 3 doses of Moderna (6,283), local reactions were reported more frequently after dose 3 than dose 2 (5,323; 84.7% and 5,249; 83.5%; p-value = 0.03) (Figure). Systemic reactions were reported less frequently after dose 3 than dose 2 (4,963; 79.0% and 5,105; 81.3%; p-value <0.001). Among registrants who received 3 doses of Pfizer-BioNTech (6,308), local reactions were reported more frequently after dose 3 than dose 2 (4,674; 74.1% and 4,523; 71.7%; p-value < 0.001). Systemic reactions were reported less frequently after dose 3 than dose 2 (4,363; 69.2% and 4,524; 71.7%; p-value <0.001). Among those who reported pain after dose 3 of an mRNA vaccine, most reactions were mild (4,909; 51.4%) or moderate (4,000; 41.9%); severe pain (defined as pain that makes daily activities difficult or impossible) was reported by 637 (6.7%).

**FIGURE:**
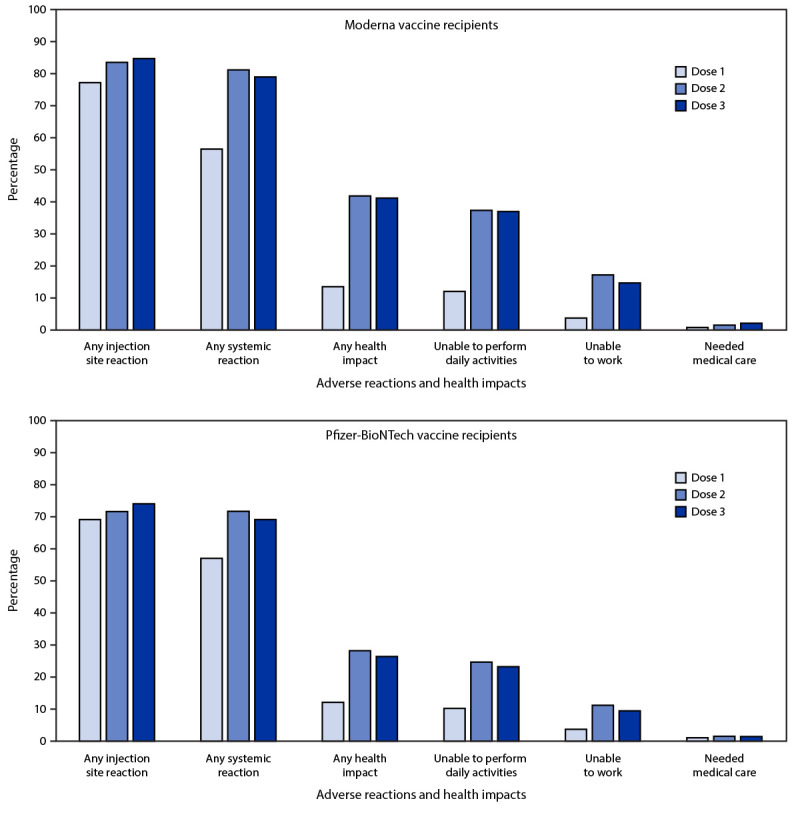
Adverse reactions and health impacts reported by persons who received 3 doses* of Moderna (N = 6,283) or Pfizer-BioNTech (N = 6,308) COVID-19 vaccine and completed at least one v-safe health check-in survey on days 0–7 after each dose, by dose number — United States, August 12–September 19, 2021 * The odds of reporting an event after dose 2 and 3 were compared using a multivariable generalized estimating equations model that accounted for the correlation between registrants and adjusted for demographic variables (receipt of care was not adjusted because of small numbers); p-values <0.05 were considered statistically significant. For Moderna recipients, all differences except any health impact and inability to perform daily activities were statistically significant. For Pfizer-BioNTech, all differences except the need for medical care were statistically significant.

## Discussion

As of September 19, 2021, approximately 2.21 million persons in the United States had received additional doses of COVID-19 vaccines^††^ after completion of a primary series. During August 12–September 19, 2021, no unexpected patterns of adverse reactions were observed among 22,191 v-safe registrants who received an additional dose of COVID-19 vaccine. Most reported local and systemic reactions were mild to moderate, transient, and most frequently reported the day after vaccination. Most registrants who received an additional dose reported a primary mRNA vaccination series followed by a third dose from the same manufacturer. The Pfizer-BioNTech clinical trial, which included 306 persons aged 18–55 years, showed that reactions after dose 3 were comparable to those reported after dose 2 (*3*). However, this analysis of v-safe data found the local reactions were slightly more common and systemic reactions less common after dose 3 of Pfizer-BioNTech. The patterns of adverse reactions observed after dose 3 of Moderna vaccine or Pfizer-BioNTech were consistent with previously described reactions after receipt of dose 2 (*4*).

The number of registrants who indicated that they received 2 doses of Janssen vaccine or received their additional dose from a manufacturer different from that of their primary series was small, limiting any conclusions. Data on the safety or effectiveness of vaccination with COVID-19 vaccine products from different manufacturers are limited; the Advisory Committee on Immunization Practices (ACIP) recommends that persons with moderately to severely immunocompromising conditions receive a third dose of mRNA COVID-19 vaccine from the same manufacturer as their primary series (*5*). CDC recommendations for an additional dose do not currently include persons who received Janssen vaccine.

During the period covered by this study, ACIP recommendations for an additional dose of COVID-19 vaccine were limited to persons with moderately to severely immunocompromising conditions who had received 2 doses of an mRNA vaccine.^§§^ A study conducted among immunocompromised hemodialysis patients reported that local and systemic reactions after dose 3 of Pfizer-BioNTech vaccine were similar to those after dose 2.^¶¶^ Recent reports of infections in vaccinated persons (*6*) and increases in the prevalence of infection with the B.1.617.2 (Delta) variant of SARS-CoV-2, the virus that causes COVID-19, among vaccinated persons (*7*) might have prompted some persons to seek an additional dose outside of recommendations. The median interval from completion of the primary series to receipt of an additional dose was approximately 6 months; therefore, persons prioritized during the rollout of COVID-19 vaccines, including health care workers and older adults, might have received an additional dose.

The findings in this report are subject to at least four limitations. First, enrollment in v-safe is voluntary and likely not representative of the vaccinated U.S. population; the majority of participants identified themselves as White and non-Hispanic. Second, during this study period, additional dose recommendations were limited to persons with immunocompromising conditions who completed a primary mRNA COVID-19 vaccination series; however, v-safe does not include information about immune status. Additional-dose recipients likely include persons with and without immunocompromising conditions. Third, a causal relationship between a vaccine and clinically serious adverse event reported after vaccination cannot be established using v-safe data. Finally, insufficient data were available to determine patterns of adverse reactions after receipt of an additional dose from a manufacturer different from the primary series or for the Janssen vaccine.

An additional dose of mRNA COVID-19 vaccine is recommended for persons with moderately to severely immunocompromising conditions (*5*). CDC recommended an additional dose of Pfizer-BioNTech vaccine ≥6 months after completion of the primary vaccine series among persons aged ≥65 years, residents in long-term care settings, and persons aged 50–64 years with underlying medical conditions; persons aged 18-49 years with underlying medical conditions and persons aged 18–64 years at increased risk for COVID-19 exposure and transmission because of occupational or institutional setting may receive an additional dose based on their individual benefits and risks (*8*). Initial analyses of safety data from >22,000 v-safe registrants shows that local reactions are slightly increased and systemic reactions are slightly decreased after dose 3 of an mRNA than after dose 2. No unexpected patterns of adverse reactions were identified; those reported were mild to moderate and transient. CDC will continue to monitor the safety of additional doses of COVID-19 vaccine. Additional data on adverse reactions associated with different combinations of vaccines and of time since completion of primary series will be important to guide public health recommendations.

**Summary**

**What is already known about this topic?**

Among 306 Pfizer-BioNTech clinical trial participants, adverse reactions after dose 3 were similar to those after dose 2.

**What is added by this report?**

During August 12–September 19, 2021, among 12,591 v-safe registrants who completed a health check-in survey after all 3 doses of an mRNA COVID-19 vaccine, 79.4% and 74.1% reported local or systemic reactions, respectively, after the third dose; 77.6% and 76.5% reported local or systemic reactions after the second dose, respectively.

**What are the implications for public health practice?**

Voluntary reports to v-safe found no unexpected patterns of adverse reactions after an additional dose of COVID-19 vaccine. CDC will continue to monitor vaccine safety, including for additional COVID-19 doses.

## Footnotes

* V-safe registrants self-report the severity of their symptoms, defined as mild (noticeable, but not problematic), moderate (limit normal daily activities), or severe (make daily activities difficult or impossible). Health impacts include whether the vaccine recipient was unable to perform normal daily activities, missed school or work, or received care (i.e., telehealth, clinic or emergency department visit, or hospitalization) from a medical professional because of new symptoms or conditions.

† Additional health surveys are sent weekly through 6 weeks after vaccination and 3, 6, and 12 months after vaccination.

§ VAERS is a passive vaccine safety surveillance system comanaged by CDC and FDA that monitors adverse events after vaccination. VAERS accepts reports from anyone, including health care providers, vaccine manufacturers, and members of the public. https://vaers.hhs.gov/reportevent.html

¶ The odds of reporting an event after dose 2 and 3 were compared using a multivariable generalized estimating equations model that accounted for the correlation between registrants and adjusted for demographic variables; p-values <0.05 were considered statistically significant.

** 45 C.F.R. part 46; 21 C.F.R. part 56; 42 U.S.C. Sect. 241(d); 5 U.S.C. Sect. 552a; 44 U.S.C. Sect. 3501 et seq.

††
https://covid.cdc.gov/covid-data-tracker/#datatracker-home

§§ On August 13, 2021, ACIP recommended an additional dose of mRNA COVID-19 vaccine after completion of a primary series in persons with moderately to severely immunocompromising conditions. Information on clinical considerations is available at https://www.cdc.gov/vaccines/covid-19/clinical-considerations/covid-19-vaccines-us.html

¶¶
https://www.medrxiv.org/content/10.1101/2021.07.02.21259913v1

## References

[1] Food and Drug Administration. Pfizer-BioNTech COVID-19 vaccine letter of authorization. Silver Spring, MD: US Department of Health and Human Services, Food and Drug Administration; 2021. https://www.fda.gov/media/150386/download

[2] Food and Drug Administration. Moderna COVID-19 vaccine letter of authorization. Silver Spring, MD: US Department of Health and Human Services, Food and Drug Administration; 2021. https://www.fda.gov/media/144636/download

[3] Pfizer Inc. Pfizer and BioNTech initiate rolling submission of supplemental biologics license applications to U.S. FDA for booster dose of Comirnaty in individuals 16 and older. New York City, NY: Pfizer Inc.; 2021. https://www.pfizer.com/news/press-release/press-release-detail/pfizer-and-biontech-initiate-rolling-submission

[4] Chapin-Bardales J, Gee J, Myers T. Reactogenicity following receipt of mRNA-based COVID-19 vaccines. JAMA 2021;325:2201–2. 10.1001/jama.2021.5374 33818592

[5] CDC. Interim clinical considerations for use of COVID-19 vaccines currently approved or authorized in the United States. Atlanta, GA: US Department of Health and Human Services, CDC; 2021. Accessed September 25, 2021. https://www.cdc.gov/vaccines/covid-19/clinical-considerations/covid-19-vaccines-us.html

[6] Brown CM, Vostok J, Johnson H, Outbreak of SARS-CoV-2 infections, including COVID-19 vaccine breakthrough infections, associated with large public gatherings—Barnstable County, Massachusetts, July 2021. MMWR Morb Mortal Wkly Rep 2021;70:1059–62. 10.15585/mmwr.mm7031e2 34351882 PMC8367314

[7] Fowlkes A, Gaglani M, Groover K, Thiese MS, Tyner H, Ellingson K; HEROES-RECOVER Cohorts. Effectiveness of COVID-19 vaccines in preventing SARS-CoV-2 infection among frontline workers before and during B.1.617.2 (Delta) variant predominance—eight U.S. locations, December 2020–August 2021. MMWR Morb Mortal Wkly Rep 2021;70:1167–9. 10.15585/mmwr.mm7034e4 34437521 PMC8389394

[8] CDC. Booster shot. Atlanta, GA: US Department of Health and Human Services, CDC; 2021. Accessed September 25, 2021. https://www.cdc.gov/coronavirus/2019-ncov/vaccines/booster-shot.html

